# Direct cyclodextrin-based powder extrusion 3D printing for one-step production of the BCS class II model drug niclosamide

**DOI:** 10.1007/s13346-022-01124-7

**Published:** 2022-02-09

**Authors:** Monica Pistone, Giuseppe Francesco Racaniello, Ilaria Arduino, Valentino Laquintana, Antonio Lopalco, Annalisa Cutrignelli, Rosanna Rizzi, Massimo Franco, Angela Lopedota, Nunzio Denora

**Affiliations:** 1 Department of Pharmacy – Pharmaceutical Sciences, University of Bari “Aldo Moro”, Orabona St. 4, 70125 Bari, Italy; 2 Institute of Crystallography-CNR, Amendola St. 122/o, 70126 Bari, Italy

**Keywords:** Three-dimensional printing, Direct powder extrusion printing, 3DForMe printer, Repositioning niclosamide, Personalized medicines, Amorphous solid dispersions, Hydroxypropyl-β-cyclodextrin

## Abstract

Niclosamide (NCS) is a drug that has been used as an anthelmintic and anti-parasitic drug for about 40 years. Recently, some studies have highlighted its potential in treating various tumors, allowing a repositioning of this drug. Despite its potential, NCS is a Biopharmaceutical Classification System (BCS) Class II drug and is consequently characterized by low aqueous solubility, poor dissolution rate and reduced bioavailability, which limits its applicability. In this work, we utilize a very novel technique, direct powder extrusion (DPE) 3D printing, which overcomes the limitations of previously used techniques (fused deposition modelling, FDM) to achieve direct extrusion of powder mixtures consisting of NCS, hydroxypropyl methylcellulose (HPMC, Affinisol 15 LV), hydroxypropyl-β-cyclodextrin (HP-β-CD) and polyethylene glycol (PEG) 6000. For the first time, direct printing of powder blends containing HP-β-CD was conducted. For all tablets, *in vitro* dissolution studies showed sustained drug release over 48 h, but for tablets containing HP-β-CD, the release was faster. Solid-state characterization studies showed that during extrusion, the drug lost its crystal structure and was evenly distributed within the polymer matrix. All printed tablets have exhibited good mechanical and physical features and a stability of the drug content for up to 3 months. This innovative printing technique has demonstrated the possibility to produce personalized pharmaceutical forms directly from powders, avoiding the use of filament used by FDM.

**Graphical abstract:**

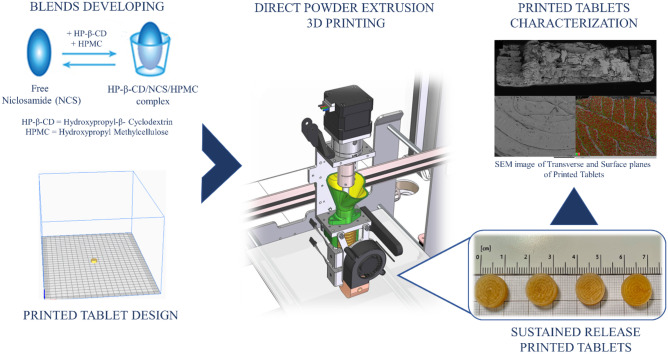

**Supplementary information:**

The online version contains supplementary material available at 10.1007/s13346-022-01124-7.

## Introduction

Niclosamide (NCS) is a drug included in the World Health Organization’s Model List of Essential Medicines [1] and has been approved in 1982 by the FDA as an anti-parasitic and anthelmintic drug. In recent years, different biological activities have been attributed to this molecule, allowing a repositioning of this drug as a good candidate for the treatment of tumor diseases [2]. Latest studies, conducted both *in vitro* and *in vivo*, have shown that NCS can act as an anticancer drug against colon, lung, breast, and prostate cancer [3, 4], and in accordance with the dose used in clinical studies [5, 6] and by applying the method of normalizing body surface area (BSA) [7], proposed by the Food and Drug Administration, the therapeutic dose of NCS is around 50 mg for a person weighing 70 kg. This drug has recently been described as a potent Stat3 inhibitor capable of suppressing Stat3 phosphorylation at Tyr705 and transcript activity [8, 9]; moreover, a correlation between Stat3 and the accumulation of myeloid-derived suppressor cells (MDSCs) in tumor-bearing mice has been demonstrated [10–12]. Treatment with NCS has been proven to reduce the number of MDSCs, which promote tumor growth and metastasis formation in breast cancer patients. Despite its potential, expectations of the NCS should be enhanced by a realistic and rational assessment, as the repositioning process requires an improvement in its chemical and physical characteristics. Indeed, NCS is a drug described by poor aqueous solubility and reduced dissolution and absorption rate [4], being a class II drug according to the Biopharmaceutics Classification System (BCS). In this regard, there are several strategies to satisfy this need, including the conversion of a drug's solid state from crystalline to amorphous [13, 14]. The use of a drug in an amorphous state involves its stabilization in a formulation, which can be obtained by generating an amorphous solid dispersion (ASD). There are numerous methods for obtaining ASD, but the most investigated one, in recent decades, is the hot melt extrusion (HME), a continuous process in which temperature and pressure are applied to soften or melt the starting material [15, 16] forcing it through an orifice and producing new products with uniform shape and density [15]. This technique produces filaments that can be utilized for the preparation of personalized dosage forms by using three-dimensional (3D) printing.

The 3D printing (3DP) is emerging as a very innovative technique capable of transforming a digital model, generated by computer-assisted design software (CAD), into a real product by the gradual layering of material [15]. For many years, 3D technologies have been employed in a broad range of research areas for the fast and cost-effective production of design models [17–19]. In the field of pharmaceutical applications, this revolutionary technology is growing in parallel with the concept of personalized medicine [20, 21], which aims to adapt medical treatments to the needs and characteristics of individual patients [22, 23]. There are many benefits that 3D printing provides including the sustainability, patentability, and the capacity to produce at lower costs [23]. In addition, 3D printing can enable a complete reorganization of galenic practice, specifically due to the small space required to set up the printing facility and extemporaneous production on demand [23]. Indeed, the real-time production offered by this technology overcomes the need for storage and the long-term stability of printed products. According to the American Society for Testing and Materials (ASTM) classification, there are seven different types of 3DP: in-tank photopolymerization, material jet, binder jet, material extrusion, powder bed casting, sheet lamination, and direct energy deposition [24]. The most widely used 3D printing technology is fused deposition modeling (FDM) [25] which allows the extrusion of starting materials, in the form of thermoplastic filaments obtained by HME, above their melting temperature. However, there are several limitations of the HME process; primarily the filament has to satisfy numerous physical and mechanical parameters to be suitable as a starting material for FDM, thus limiting the choice of active compounds and excipients [26]. In addition, there may be complications with filament extrusion [27], especially when high drug dosages are requested, or degradation of active compounds due to high melting temperatures that may reduce the drug loading and release profile [24, 26]. The possibility to overcome all these limitations is offered by direct powder extrusion (DPE) 3DP. In fact, through this pioneering technology, it is possible to extrude material directly from pellets or powder using a single-screw extruder [24], so preparation of the filament by HME is not necessary and it is possible to extrude mixtures that could not be extruded by FDM. In this field, it is critical to include the detailed study of the excipients to be employed, in order to assess their effective extrudability as powder by means of DPE. The extrudability of blends of three different excipients, namely hydroxypropyl methylcellulose (HPMC, Affinisol 15 LV), hydroxypropyl-β-cyclodextrin (HP-β-CD) and polyethylene glycol (PEG) 6000, was evaluated. The extrusion of cyclodextrin (CD) by HME to enhance the solubility of class II BCS drugs is well documented [26, 28]; nevertheless, the direct extrusion of CDs by DPE has not been explored so far. CDs have been widely used as pharmaceutical grade excipients for many years, yet they are still very innovative due to their remarkable properties, including the ability to form inclusion complexes with poorly soluble drugs, thereby increasing their solubility [29, 30]. Therefore, repurposing NCS at therapeutically relevant dosages as a drug for the treatment of tumor diseases, and to overcome the limitations of its low solubility and poor dissolution and absorption rate, in this study sustained-release printed tablets by DPE have been developed using HPMC as a carrier polymer, HP-β-CD as a solubilizing and complexing agent and (PEG) 6000 as a plasticizer. Since the use of a hydrophilic polymer could facilitate the stabilization of a drug/CD complex [30–32], the formation of a ternary HPMC/HP-β-CD/NCS complex has been explored.

The aim of this study was to explore the use of an innovative technology, the 3DP DPE, highlighting the ability, for the first time, to directly extrude powders containing HP-β-CD, and to overcome the solubility limitations associated with NCS (class II BCS drug). Precisely, this aspect has represented the highly ground-breaking idea behind this work, which for the first time has focused attention on the considerable potential that the HP-β-CD could bring even in the application of the DPE 3D technique. Accordingly, NCS ASDs were manufactured as sustained release printed tablets from powder mixtures with different compositions. Solid-state characterization studies were carried out on the various mixtures and formulations obtained, as well as the study of drug dissolution profiles and evaluation of the stability of the 3D printed tablets.

## Materials and methods

### Materials

Niclosamide and sodium hydroxide were purchased from Sigma-Aldrich—Merck (Darmstadt, GERMANY). Hydroxypropyl-β-cyclodextrin, (Cavasol W7, HP-β-CD with MW = 1540, molar substitution degree SD = 7) and Polyethylene Glycol 6000 and Micronized Talcum Powder (Ph. Eur. E 553b) were purchased from Farmalabor (Canosa di Puglia, ITALY). AFFINISOL™ HPMC HME 15 LV (hydroxypropyl methylcellulose) was gifted by Pharma Solutions – Nutrition & Biosciences Italy (Milano, ITALY). Aerosil 200 micronized silica was purchased from Fagron (Rotterdam, NETHERLANDS). Ethanol 96% vol was purchased from VWR Chemicals Italia (Milano, ITALY). Polysorbate 80 and Potassium Phosphate Dibasic were purchased from Honeywell Fluka Italia (Rodano, ITALY). For the analysis, distilled and purified water (conductivity of 18.2 MΩ.cm at 23 °C) was obtained by the purification system Milli-Q (Purelab DI, MK2) (Elga, High Wycombe, UK).

### Quantitative analysis of NCS

For the quantitative analysis of NCS, both UV and HPLC analyses were performed. PerkinElmer double-beam UV–visible spectrophotometer Lambda Bio 20 (Milan, Italy) was used, equipped with 10 mm path-length-matched quartz cells. Standard calibration curves were prepared at a wavelength of 340 nm using ethanol as solvent. A calibration straight line (R2 = 0.9999), in the concentration range between 0,305 µM and 152,5 µM, was obtained. To qualitatively assess the presence of not degraded NCS during the printing process, HPLC analyses were conducted. A sample of NCS maintained at 180 °C for 15 min and samples derived from the solubilization of formulation 4 (see below) were also analyzed. The investigations were performed with an Agilent 1260 Infinity Quaternary LC System equipped with an Agilent variable wavelength UV detector, a Rheodyne injector (Rheodyne, Model 7725i) equipped with a 20 µL loop and a OpenLAB CDS ChemStation software (Agilent, Santa Clara, CA). A ZORBAX Eclipse plus C18 column (4.6 × 250 mm 5-Micron) was used as the stationary phase and maintained at 30 °C for the duration of the analysis. The mobile phase consisted of methanol/phosphate buffer 1 mM (85:15 v/v) at pH 5.47 and was pumped at a flow rate of 0.7 mL/min. The wavelength selected for elution monitoring was 334 nm. A calibration line (R2 = 0.9998) was obtained by analyzing a range of concentrations between 0.05 and 0.005 mg/ml. Samples were further analyzed by mass spectrometry performed using Agilent 6530 accurate mass Q-TOF. Mass spectra were achieved in negative (ESI-).

### Preparation of powder blends

Four samples constituted of polymer powder blends at different concentrations and a constant drug concentration (NCS 10% w/w) were prepared. In each mixture, a cellulosic matrix, such as Affinisol HPMC HME 15 LV, was used in added PEG 6000 (blends 2 and 4) as plasticizer and, HP-β-CD (blends 3 and 4) to improve drug solubility were employed. The final composition of each mixture is shown in Table 1.

**Table 1:** Composition of powder mixtures

**Samples**	**NCS**	**HPMC**	**PEG 6000**	**HP-β-CD**
	(% w/w)			
**Blend 1**	10	90.00	/	/
**Blend 2**	10	85.50	4.50	/
**Blend 3**	10	42.87	/	47.13
**Blend 4**	10	40.73	2.14	47.13

Each component was sieved three times through a 355 µm mesh sieve to provide better dimensional uniformity and mixing of the powders. Next, the powders were mechanically stirred for approximately one hour at 67 rpm using the Turbula Willy A. Bachofen GmbH (Nidderau, Germany). The resulting powder blends were dried overnight at 40 °C in stove.

### Solid state characterization of powder mixtures

The four powder blends were characterized in the solid state together with the pure drug and the physical blends consisting only of the excipients. They were studied by Fourier transform infrared spectroscopy (FT-IR), differential scanning calorimetry (DSC), thermogravimetric analysis (TGA) and powder X-ray diffractometry (PXRD). For FT-IR analysis, KBr pellets (2% of sample) were analyzed by FT-IR 1600 PerkinElmer spectrophotometer. Data were acquired between 4000 cm−1 and 400 cm−1. Thermal analyses by DSC of different samples were performed by a Mettler Toledo DSC822 instrument. About 5–10 mg of sample were heated in an aluminum pan with a 5 °C/min heating rate from 25 °C to 280 °C under N2 flow. An empty pan was used as a reference. The TGA analysis were performed on Blend 4 using PerkinElmer Thermogravimetric Analyzer Pyris 1 TGA. Sample (9 mg) was placed in platinum pans and was then heated from 30 to 600 °C using 5 °C/min as a heating rate. The thermal decomposition (or degradation) profile was analysed using Pyris™ software version 11. The experiments were carried out under nitrogen gas flow of 20 mL/min. The diffraction patterns were collected by using a Rigaku Rint2500 rotating Cu anode, working at 50 kV and 200 mA in Debye − Scherrer geometry. The diffractometer is equipped with an asymmetric Johansson Ge (111) crystal to select the monochromatic CuKα1 radiation (λ = 1.54056 Å) and the silicon strip Rigaku D/teX Ultra detector. The range from 5 to 90° (2ϑ) was collected with a 0.02° (2ϑ) step size and counting time of 6 s/step. Each powder was introduced in a glass capillary of 0.5 mm of diameter and mounted on the axis of the goniometer. The capillary was rotated during the measurement to improve the randomization of the orientations of the individual crystallites to reduce the effect of possible preferred orientation.

### Phase solubility studies

The phase solubility study of NCS with HP-β-CD was performed following the Higuchi-Connors method [33]. 2 mL samples containing solutions at various concentrations of HP-β-CD were prepared, covering a concentration range from 0.0023 to 0.26 M. An excess amount of drug was placed inside each solution, and the resulting suspensions were sonicated for 2 min at 37 °C. Subsequently, they were allowed to rest within a thermostatic bath at 37 °C at a constant oscillation for 48 h. After reaching equilibrium, the samples were centrifuged at 10,000 rpm for 15 min and the supernatant filtered through 0.45 µm cellulose acetate (CA) membrane filters was analyzed. The concentration of NCS was assessed by UV at a wavelength of 340 nm after 1/10 dilutions in ethanol, previous development of the NCS calibration line. The solubility diagram was obtained by plotting the drug molar concentration against the HP-β-CD molar concentration. Calculation of the complexation constant (Kc1:1) was performed using Eq. (1) [33]:

1 $$Kc1:1=\frac{P}{{S}_{0}\; \left(1-P\right)}$$

where:

$$\mathrm{S_0}=\mathrm{Intrinsic \;solubility \;of\; NCS \;in \;water}.$$

$$\mathrm{P}=\mathrm{ Slope\; of \;the \;phase \;solubility \;diagram}.$$

Moreover, the phase solubility study was carried out in the presence of CD (different concentrations from 0.00325 M to 0.026 M) and 40% w/w HMPC (same HP-β-CD/HPMC ratio in blends 3 and 4), in order to assess whether the formation of a ternary polymeric system could generate a further increase in drug solubility. The same procedure as described above was performed.

### Homogeneity of HME filaments

**Fig. 1:**
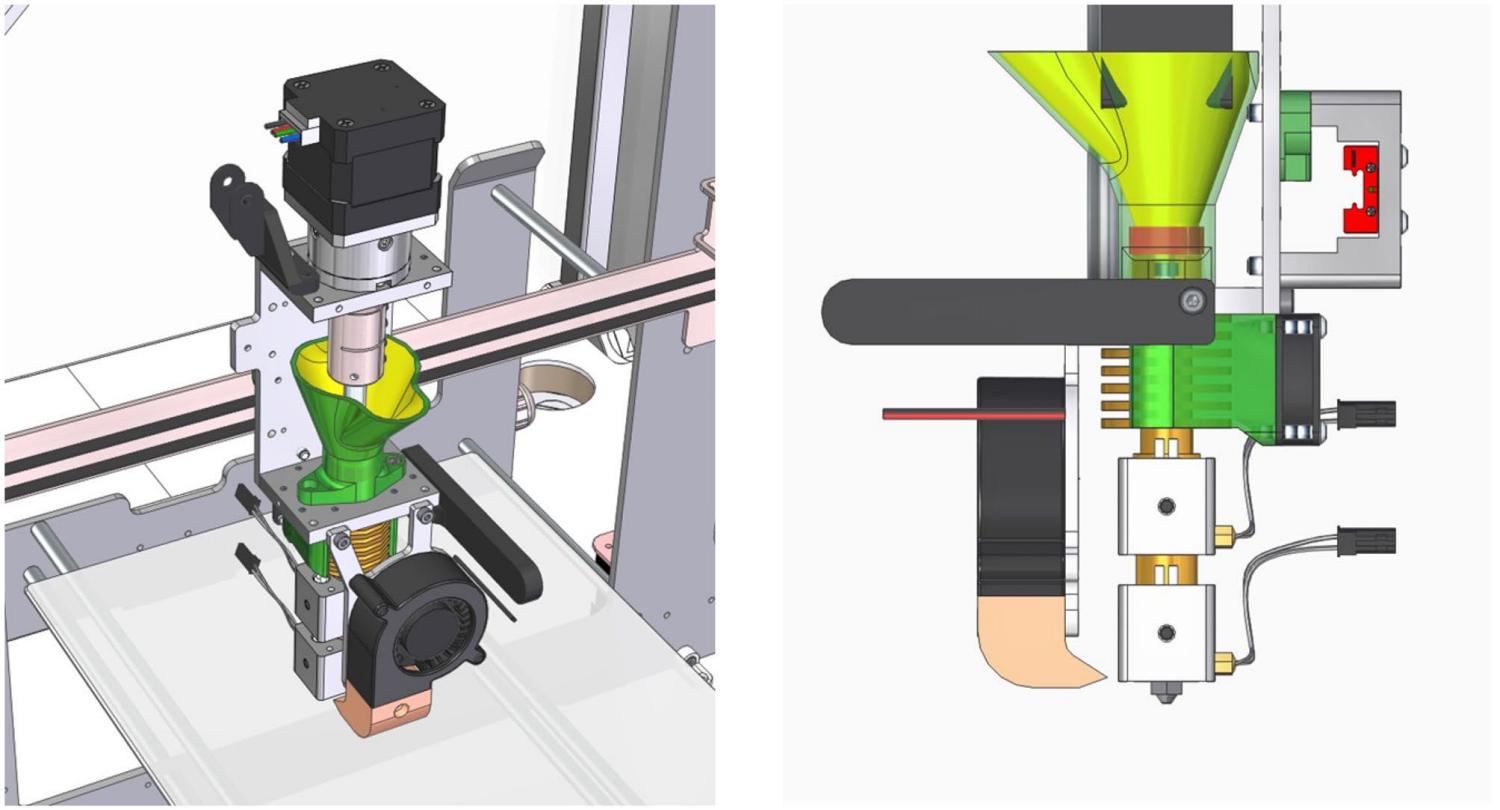
Design of the nozzle of the direct single-screw powder extruder Farmalabor 3D printer

Before the printing stage, preliminary studies were performed to evaluate the behavior of the four blends during extrusion as well as the characteristics of the extruded filament. In the present study, the 3DForMe® 3D printer (Farmalabor, Canosa, ITALY) was used (Fig. 1), consisting of a loading hopper, a single-screw extruder and temperature sensing and control systems. 2 g of each blend were placed in the hopper and the formation of the filament by the extrusion was operated at 180 °C. At the end of each extrusion the filaments were weighed to calculate the yield of the process. Then, from each generated filament, 5–8 pieces of 2 cm length were cut, exactly weighted, and studied to demonstrate the homogeneity of the printed filaments. In particular, visual inspection and amount of NCS were considered. The pieces were dissolved in 4 mL of ethanol by stirring overnight. After appropriate dilution, the solutions were analyzed by UV to calculate di amount of NCS in each piece.

### Direct powder extrusion 3D-printing

The tablets were printed using 3DForMe® 3D printer (Fig. 1), which is specifically designed for pharmaceutical manufacturing. The model of the tablets was created using the CAD software Fusion360, which allowed the creation of stereolithography (.stl) files that were subsequently exported to the 3D printer software (Ultimaker Cura). The stereolithography file describes the geometry of the object, while all other parameters are set directly in the Ultimaker Cura software. A cylindrical geometry was selected for the tablet 3D printing with dimensions of 12 mm diameter × 3.7 mm height. This size made it possible to obtain tablets of the required weight, 500 mg. The parameters set in the software for printing were: Infill 70% with infill pattern concentric, top and bottom pattern zig-zag, high resolution with brim, without raft, travel speed 5 mm/s, print speed 5 mm/s, number of shells 2, layer height 0.2 mm, floor temperature 70 °C, and extrusion temperature of 180 °C. The prepared powder mixture was added to the extruder hopper of the 3D printer, which used was specifically designed with a direct single-screw powder extruder and a nozzle diameter of 0.8 mm. The extruder design is based on a single screw HME, and the rotation speed was controlled by the 3D printer software. Furthermore, the extruder nozzle moves in three dimensions to create the objects in a layer-by-layer texture. During printing, all blends met the same extrusion temperature conditions set in the software. At the end of each formulation printing, the extruder is disassembled and removed from the screw, which is cleaned to avoid contamination between the blends studied.

### Characterization of printed tablets

The physical dimensions of the printed tablets were assessed by a digital slide gauge Hitech Diamond. To evaluate the weight uniformity, 10 tablets of each formulation were randomly selected and accurately weighed. Following, the mean weight was calculated, and this value was compared with each of the 10 weights verifying that all tablets had a weight respecting the standard deviation of ± 10% w/w from the mean, as required by the quality controls for magistral preparations reported in F.U. I. XII [34]. The morphology of the tablets obtained at the printing stage was evaluated by electrical scanning microscopy (SEM), operating at 20 kV (Hitachi TM 3000 Tabletop SEM). Furthermore, a chemical microanalysis test was conducted on the four tablets to confirm the presence of N and Cl, elements present only in the NCS structure and thus investigate the dispersion of the drug within them. The surface of each printed tablets was analyzed using Swift ED3000 Oxford Instrument with AZtecOne software. The breaking strength of 10 tablets of each formulation type was evaluated using a conventional Erweka GmbH D-63150 durometer. Then, an increasing force was applied to each tablet perpendicular to the plane until a fracture was formed on the tablet surface, thus indicating the total breaking force value relative to the individual tablet. The friability test was performed on the printed tablets as reported in F.U. I. XII for tablets having a mass less than or equal to 650 mg [35]. Thus, a number of tablets, having a total mass as close as possible to 6.5 g, were properly depulverized, weighed and then placed inside a friabilometer (Erweka GmbH TA 100) rotating at 25 ± 1 rpm. The test was conducted for 4 min, at the end the recovered tablets were again dusted and weighed. Weight differences were calculated and expressed as a percentage of the original sample weight. The tablets obtained were subjected to the disaggregation test as described in F.U. XII [36]. A basket disruptor (Erweka ZT3-1 Disintegration Tester) was therefore used for the analysis. The tablets, placed in the appropriate baskets, were responded for 2 h in 0.1 M hydrochloric acid without the use of discs and then the acid medium was replaced with a solution of phosphate buffer pH = 6.8 for 60 min with the addition of discs inside the baskets.

Finally, 10 tablets of each formulation were dissolved in ethanol to evaluate the actual loading of NCS. In detail, each tablet was placed in 100 mL of ethanol and stirred overnight at room temperature. Finally, after appropriate dilution, the drug concentration was assessed by UV and HPLC analysis as above reported.

### Solid state characterization of tablets

Each formulation was characterized by FT-IR, DSC and PXRD, and the formulation 4 was also analyzed by TGA. The procedures adopted have been previously described in the section on solid state characterization of the powder mixtures. Prior to analysis, the different printed tablets were fragmented, ground, and sieved.

### Dissolution testing

The dissolution profiles of the NCS loaded in the tablets were studied for 48 h using the VK 7000 paddle dissolver. Tablets were placed in vessels containing 900 mL of simulated gastric fluid (0.2% w/v NaCl and 2% w/v Tween 80 in 0.1 N HCl, pH 1.2) for the first 2 h. For the subsequent hours, analysis was performed by placing the sample in 900 mL of simulated enteric fluid (2% w/v Tween 80 in phosphate buffer pH 6.8, F. U. I. XII [37]). Dissolution was performed at 37 °C under paddle stirring (100 rpm). NCS quantitative evaluation was conducted by taking hourly 5 mL samples from the solution and assessing the concentration of NCS dissolved by UV.

### Stability studies

The samples were stored in a Climacell 222 – ECO line climatic chamber (MMM Group, Semmelweis Strasse, München, Germany) at 25 °C and 60% relative humidity (RH) for 3 months. The printed tablets were packaged in amber glass bottles and closed with plastic screw caps. They were monitored by DSC analysis and content of NCS over the period of storage.

### Statistical analysis

The experimental data were reported as mean ± SD (standard deviation). Statistical analysis was conducted by Graph Prism version 6.0 (GraphPad Software Inc., La Jolla, CA, USA).

## Results and discussion

This work presented and used for the first time a new printer, 3DForMe®, which is based on the ability to extrude powders directly, thus overcoming the filament preparation problem associated with FDM. Different blends consisting of NCS and various concentrations of HPMC, HP-β-CD and PEG 6000 were first characterized and then extruded as such through 3DForMe®, obtaining sustained release tablets. A complete solid-state characterization of the different blends and tablets achieved has been described, together with *in vitro* dissolution studies of the printed tablets.

### NCS Phase solubility studies

**Fig. 2:**
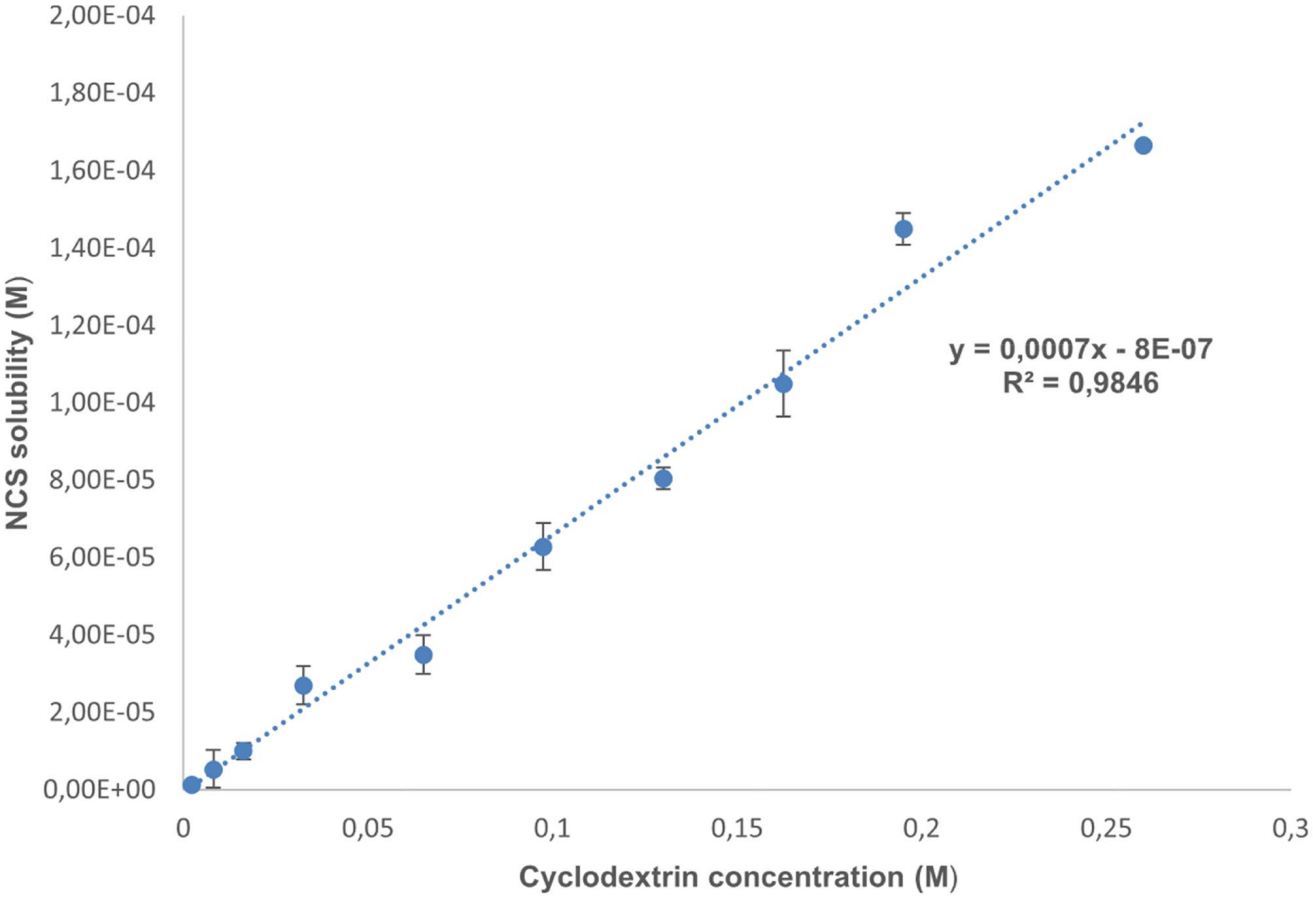
Phase solubility diagram. The change in NCS solubility within solutions of increasing HP-β-CD concentration (0.0023—0.26 M) was assessed. The analysis was carried out in triplicate

Phase solubility studies of the drug in the presence of HP-β-CD were performed, allowing the evaluation of the stability constant and to foresee the stoichiometric ratio of the NCS- HP-β-CD complex. Pure NCS has an intrinsic solubility of 8 µg/mL in water. The phase solubility plot displays a significant and linear increase in NCS solubility with increasing HP-β-CD concentration (Fig. 2). The drug shows a solubility of 54.46 µg/mL in the presence of HP-β-CD at a concentration of 0.26 M. The regression analysis (R2 = 0.9846) describes an AL-type curve, according to the Higuchi-Connors classification, with a slope of less than 1, which could be associated with a 1:1 molar ratio between NCS and HP-β-CD. By Higuchi Connors equation was calculated the constant stability equal to 876.1 M−1 indicating a good interaction between NCS and HP-β-CD. Indeed, in literature stability constant values between 50–2000 M−1 are regarded favorable [38].

**Fig. 3:**
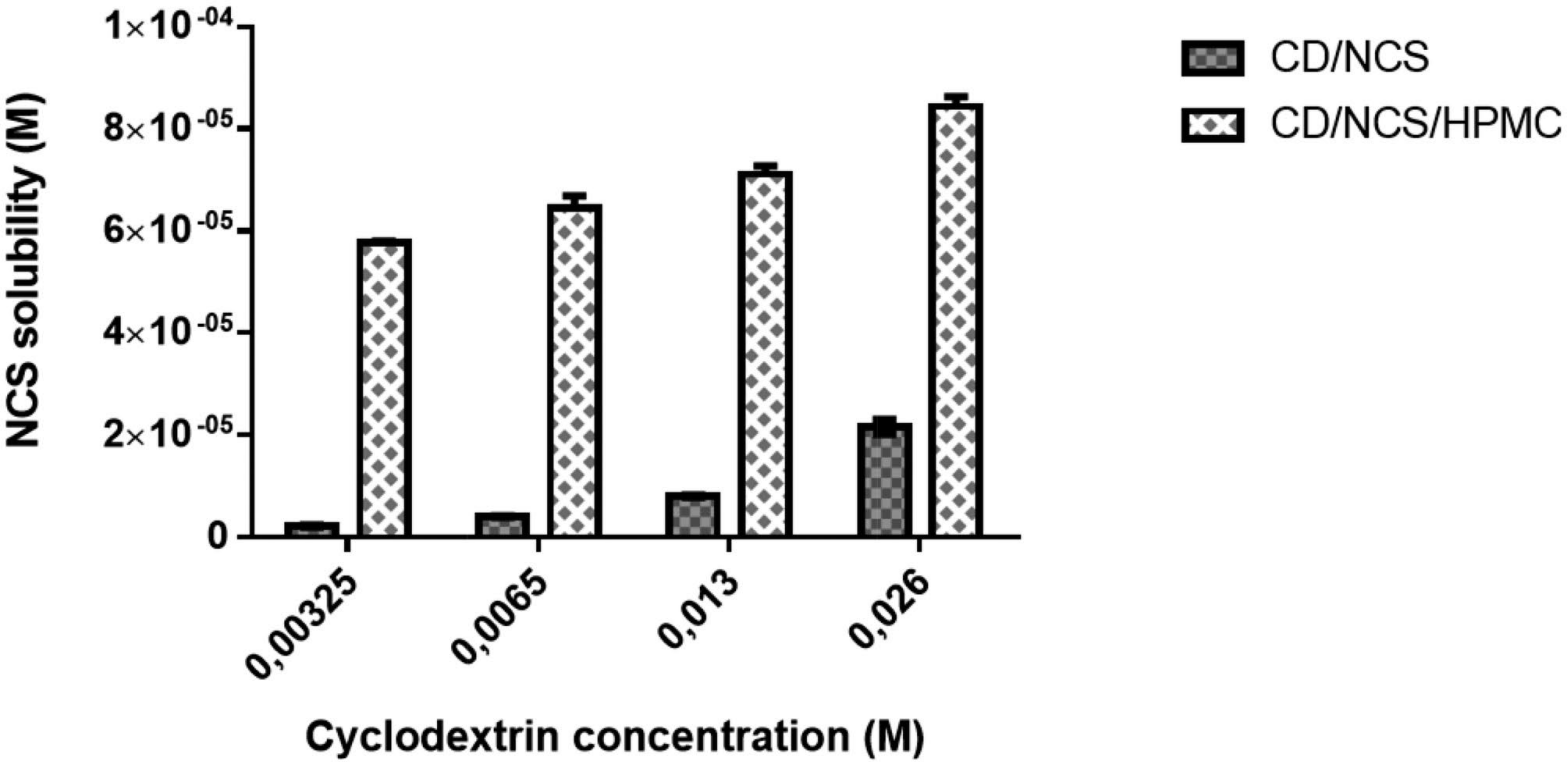
Aqueous solubility of NCS in the presence of binary (CD/NSC) and ternary (CD/NCS/HPMC) polymeric systems at different HP-β-CD concentrations. The analysis was conducted in triplicate

The addition of HPMC at HP-β-CD solution significantly improved the aqueous solubility of NCS, confirming that the use of hydrophilic polymers such as HPMC increased the CD complexation efficiency for drugs [31]. In all samples containing the ternary system (HPMC/HP-β-CD/NCS), a tenfold increase in aqueous solubility of NCS was observed in comparison to solution containing the HP-β-CD (Fig. 3). Based on these results, it could be stated that HPMC and HP-β-CD showed a synergistic effect in enhancing NCS solubility.

### Visual inspection and homogeneity of HME filaments

**Fig. 4:**
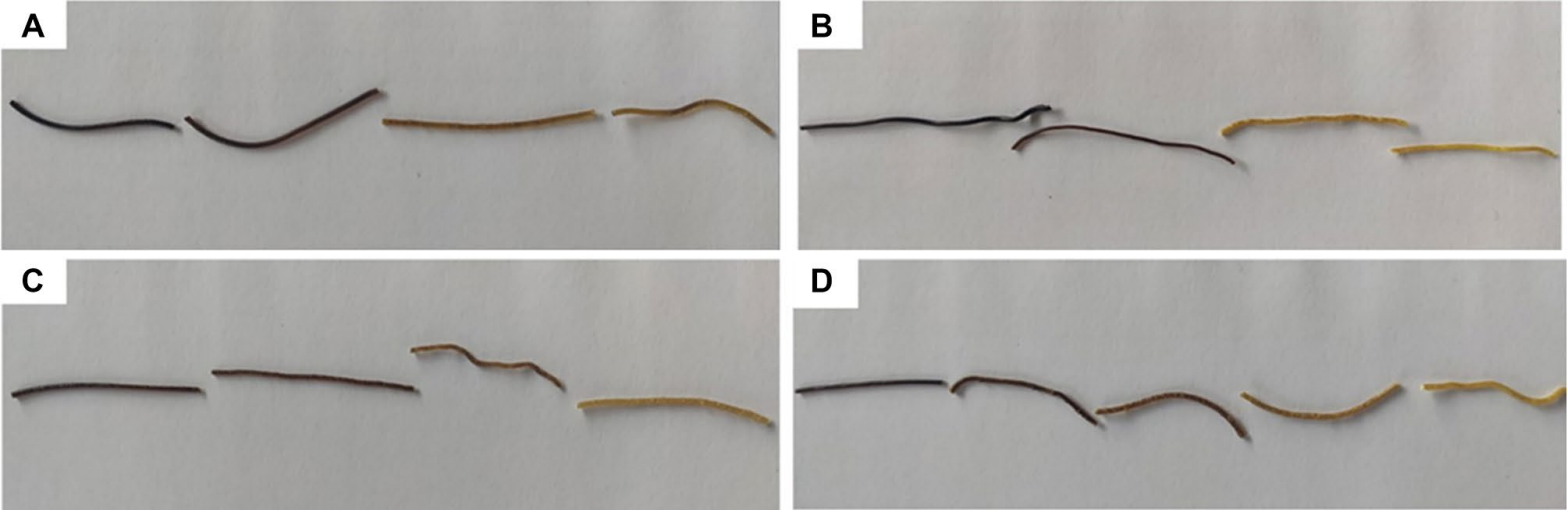
Fragments derived from a single filament: **A** Filament 1; **B** Filament 2; **C** Filament 3; **D** Filament 4

The filaments share some common characteristics, even though they are derived from different powder blends. In fact, all four filaments show a common color variation during extrusion: the filament at the beginning of the extrusion has a very dark color that tends to lighten at the end of the process (Fig. 4). These variations in terms of color are most probably due to the different time the powder mixtures were kept in the extrusion head. The four filaments had a smooth surface and were not brittle at handling.

The yield of the extrusion process was very high, considering that almost all the powder in the hopper was completely extruded and that the weight of the entire filament coincided with the weight of the blend from which it was obtained.

**Fig. 5:**
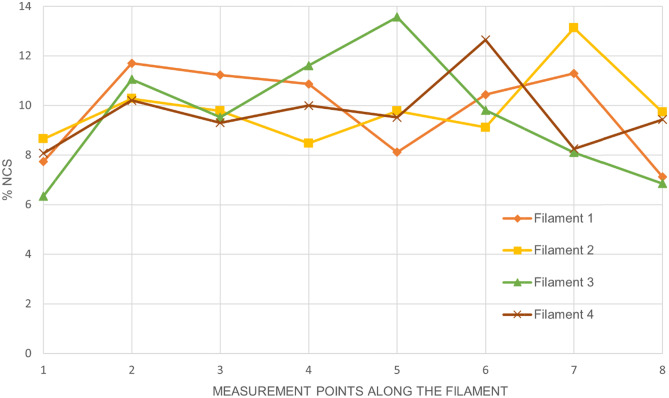
Homogeneity study of HME filaments. The graph shows the concentration (%p/p) of NCS as a function of the measuring points. Each point corresponds to the fragment obtained from the filament in accordance with the extrusion order. The analysis was carried out in triplicate

Each piece of filaments, cut in equal length, had a similar weight and although the change in color of the extruded filament might suggest degradation or a change in the stability of the drug, which often occurred during the HME process, the NCS present within the entire filament obtained with the DPE technique retained its stability (see Table 2). Indeed, respecting the correct extrusion order, the single pieces were analyzed to assess the concentration of NCS present in them. The study of filament homogeneity shows that in the four filaments there are fragments in which the drug is under-extruded, which are compensated by fragments in which the drug is over-extruded (Fig. 5). For all the filaments, it can be seen that the values of the percentage of drug are in a range between 6.34 and 13.57 (% w/w). These results are in agreement with the literature [39], as Vidik et al. justified the presence of under-extruded and over-extruded fragments as a consequence of demixing of the raw materials during the printing process. However, unlike the data reported by Vidik et al. in which the average drug concentrations in the different filament fragments were lower than the theoretical value, in the present study the average of the values obtained is very close to the value of the theoretical concentration. The results demonstrated that the adopted process was not detrimental for the drug (Table 2) despite the contact with the high temperatures of the extruder.

**Table 2:** Average of NCS concentrations (%) present within the four different HME filaments

Filament	Theoretical NCS (% w/w)	Measured NCS (% w/w) *
1	10	9.81 ± 1.72
2	10	9.87 ± 1.36
3	10	9.61 ± 2.30
4	10	9.68 ± 1.32

*The value is the average of 8 fragments. ± is the deviation standard

### Direct powder extrusion 3D-printing

The single screw extruder printer model was used for the first time to enable DPE. The blend powder realized was inserted in the hopper, heated to a specific temperature, and used for direct tablet printing. This innovative printing technique overcomes the limitations associated not only with conventional tablet printing systems [19], but also with established printing processes such as HME. The elimination of the preliminary HME step followed by FDM printing makes the DPE printing process much simpler and faster. In fact, the overall process of printing one tablet takes 13 min. In addition, with no intermediate steps, the amount of raw material waste is also greatly reduced. The design of the extruder with a vertical orientation and its adequate distance from the hopper facilitate the flow of powder into the screw. A functional amount of powder can be placed inside the hopper to ensure a production from 1 to 15 tablets for cycle. This makes the 3D printer suitable for formulating personalized medicine often required with galenic preparations.

**Fig. 6:**
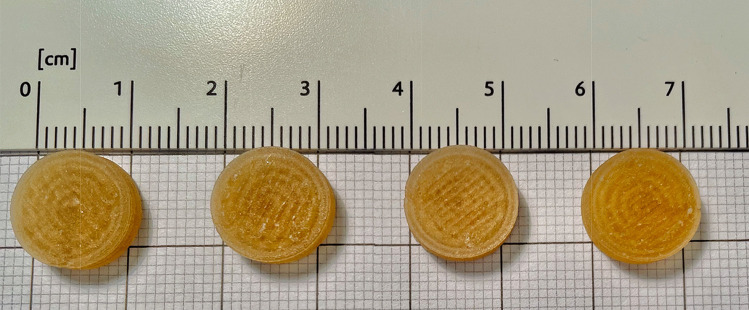
From left to right: Formulation 1, Formulation 2, Formulation 3 and Formulation 4 derived from the extrusion of Blend 1, 2, 3 and 4 respectively

All powder blends were found to be suitable for DPE 3DP. Only the extrusion of powder blend 3 led to the formation of final tablets considered unsatisfying. Different temperatures and printing parameters (screw speed) were tested in order to improve the characteristics, but none of the modifications improved the printing quality. A possible cause may be attributable to the presence of cyclodextrin, which tended to moisten the powder as the residence time in the printer increased, creating vacuum zones along the screw, and leading to extruder blockage. This problem did not occur during the filament extrusion phase due to the reduced time the powder spent in contact with the extruder. This demonstrates that the DPE requires that the powders must have a certain degree of fluidity and homogeneity to ensure that the flow through the extruder is uniform [40]. Blend 3 was then modified by adding 2% silica and 1% talc, as dehydrating and glidant agents, respectively, which allowed printing without any problems It should be noted that Blend 4 also consisted of a percentage of HP-β-CD; however, the presence of the plasticizer and glidant agent (PEG 6000) helped the printed process, favoring the powder to flow within the screw. The resulting NCS printed tablets showed a cylindrical shape and good adhesion between the printed layers (Fig. 6).

### Characterization of tablets

The tablets showed good uniformity in physical dimensions and high efficiency in reproducing the CAD drawing, which was set at 12 mm in diameter and 3.7 mm in height. The printed tablets had an average diameter ranging from 11.63 mm to 12.15 mm, and the average height ranged from 3.49 mm to 3.74 mm. This accuracy in the printing of the tablets was confirmed by data reported in the literature obtained with the same printing method [41, 42]. The average mass was described as ranging from 414.00 to 506.80 mg, this variation could be due to the different composition of the individual blends, which gives the powders different flow properties in the extruder. Despite these differences, all formulations satisfied the weight uniformity assay performed, showing weight deviations from the mean value congruent with the required acceptance limits (± 10%) [34]. All tablets had functional mechanical properties for packaging and handling. The required breaking load of the tablets has values above 200 N up to the maximum value measurable with the durometer, 484 N. The strength of the printer tablets was also confirmed by the friability test conducted, which was satisfied as each formulation had a weight loss significantly less than 1%, which is indicated by F.U.I. XII as the maximum acceptable value [35]. From the content uniformity test, the concentration of drug present in 10 tablets of each formulation was obtained, confirming the closeness to the theoretical value of the drug, and demonstrating the absence of drug degradation during extrusion. All the data described are shown in Table 3.

**Table 3:** Characteristics of formulations printed by DPE

Formulation	Weight Uniformity* (mg)	Drug content* (%)	Friability* (%)	Breaking Force (N)*	Dimensions*	
					Diameter (mm)	Height (mm)
Formulation 1	414.00 ± 26.84	9.74 ± 1.85	0.060	416.80 ± 61.27	11.63 ± 0.20	3.74 ± 0.06
Formulation 2	480.30 ± 24.41	9.86 ± 0.50	0.000	484.00 ± 0.00	12.15 ± 0.24	3.66 ± 0.23
Formulation 3	506.80 ± 28.04	10.42 ± 0.37	0.099	429.40 ± 94.82	12.15 ± 0,65	3.49 ± 0,31
Formulation 4	494.70 ± 28.16	10.23 ± 0.40	0.038	226.40 ± 99.18	12.09 ± 0.75	3.65 ± 0.15

*The value is the average of 10 tablets. ± is the deviation standard

Regarding the disaggregation test, as expected no formulations showed signs of disaggregation or breakage at the end of the test. In fact, the 3D printed tablets were intact after both acid and basic media exposure. Therefore, the results confirmed that these pharmaceutical dosage forms did not disaggregate, but they released the loaded drug following swelling of the matrix and solubilization of the excipients.

**Fig. 7:**
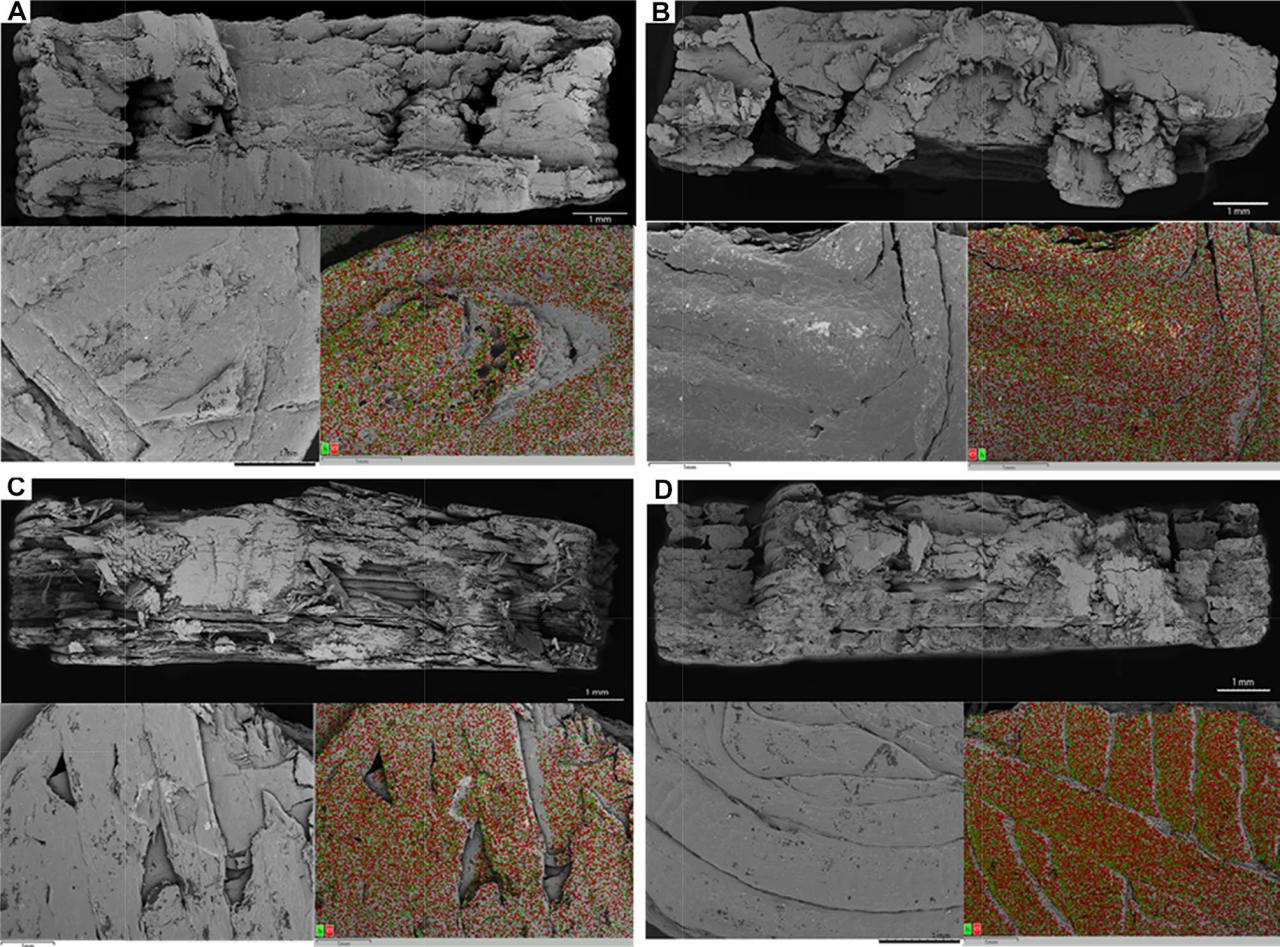
SEM images of the cross section (top in figures) and surface (bottom left in figures) and surface chemical microanalysis (bottom right in figures) of the samples: Formulation 1 **A**, Formulation 2 **B**, Formulation 3 **C**, and Formulation 4 **D**

SEM images show the surface and transverse planes of the four different formulations obtained (Fig. 7). Although the tablets were derived from blends consisting of different excipients at various concentration, they are morphologically similar. In the cross section, the three-dimensional layer-by-layer structure, characteristic of tablets obtained by 3D printing, can be seen. Each layer has a thickness of 0.2 mm, as determined by the printing parameters. The surface plane shows the concentric geometry of the chosen infill.

The data obtained from the chemical microanalysis show a homogeneous presence of the elements N (green in Fig. 7) and Cl (red in Fig. 7), which, being atoms exclusively present in the structure of the drug, indicate an equal distribution of NCS within the printed tablets.

### Solid state characterization of printed tablets

**Fig. 8:**
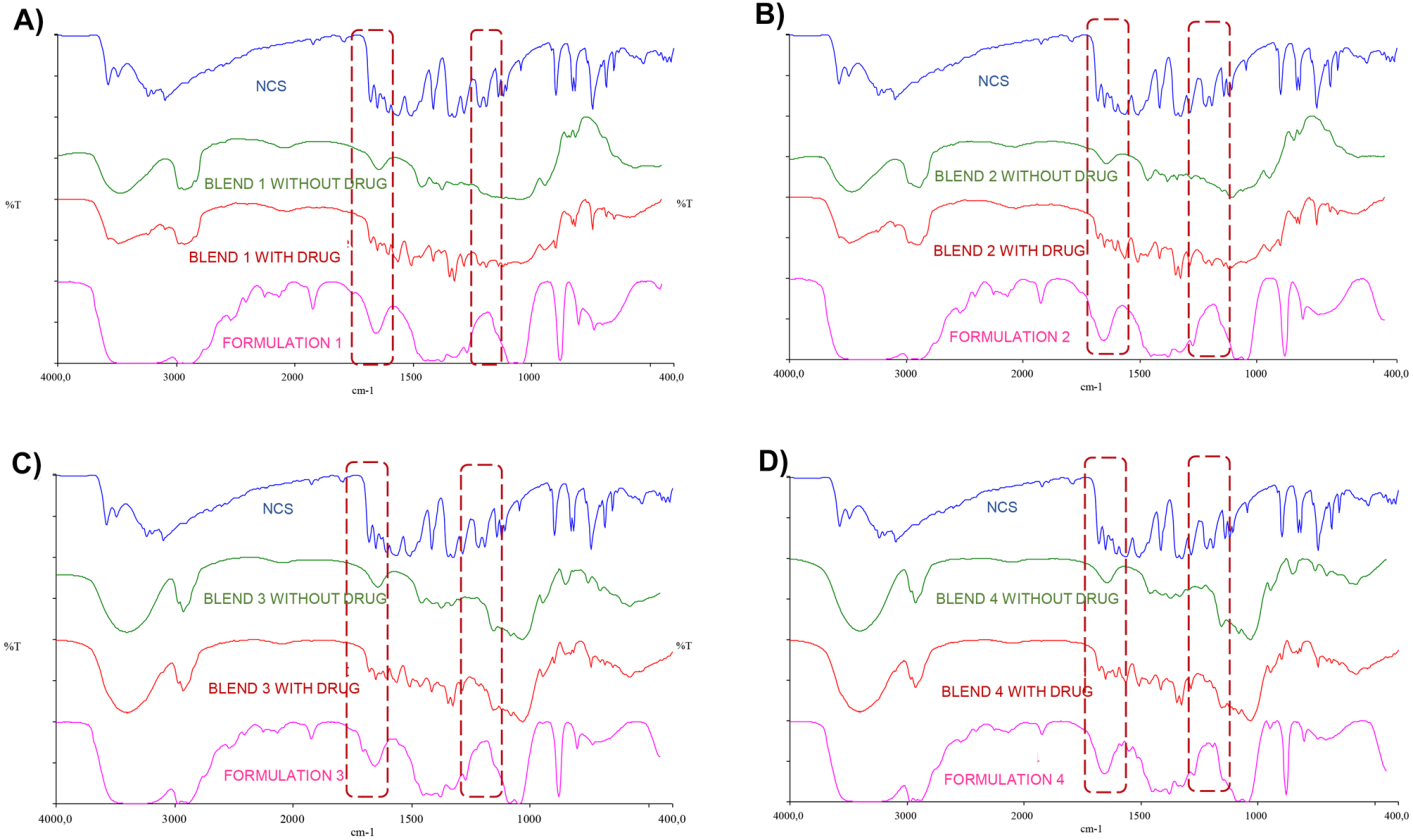
FT-IR spectra of formulations 1 **A**), 2 **B**), 3 **C**), 4 **D**), each compared with the spectra of the NCS and the respective blends with and without drug

The possible amorphization process undergone by the drug during extrusion was verified by solid-state characterization studies of the printed tablets. Using FT-IR it was observed that characteristic peaks in the NCS spectrum were present at 1218, 1520, 1570 and 1650 cm−1 (Fig. 8). The presence of these peaks was confirmed in the blend with the drug spectrum for each formulation. Peaks around 3300 cm−1 are characteristic of HP-β-CD as seen in the blends with and without the drug (Fig. 8 C-D). From the analysis of the spectrum relative to the formulations obtained from the above blends, we can see a relative widening of the HP-β-CD peak in a range between 3350 and 3100 cm−1. This widening could be reasonably attributed to the interaction of HP-β-CD with HPMC and NCS during the printing step [26]. The same observation can be made regarding the NCS peaks between 1600 cm−1 and 1800 cm−1 and between 1250 cm−1 and 1100 cm−1. The absence of important peaks characteristic of NH stretching and the stretching of the carbonyl group of the drug could suggest the possible amorphization of the drug inside the printed tablets and/or the interaction between the drug and the HP-β-CD [4]. These results could confirm the formation of intimate complexes of HP-β-CD, NCS and HPMC.

**Fig. 9:**
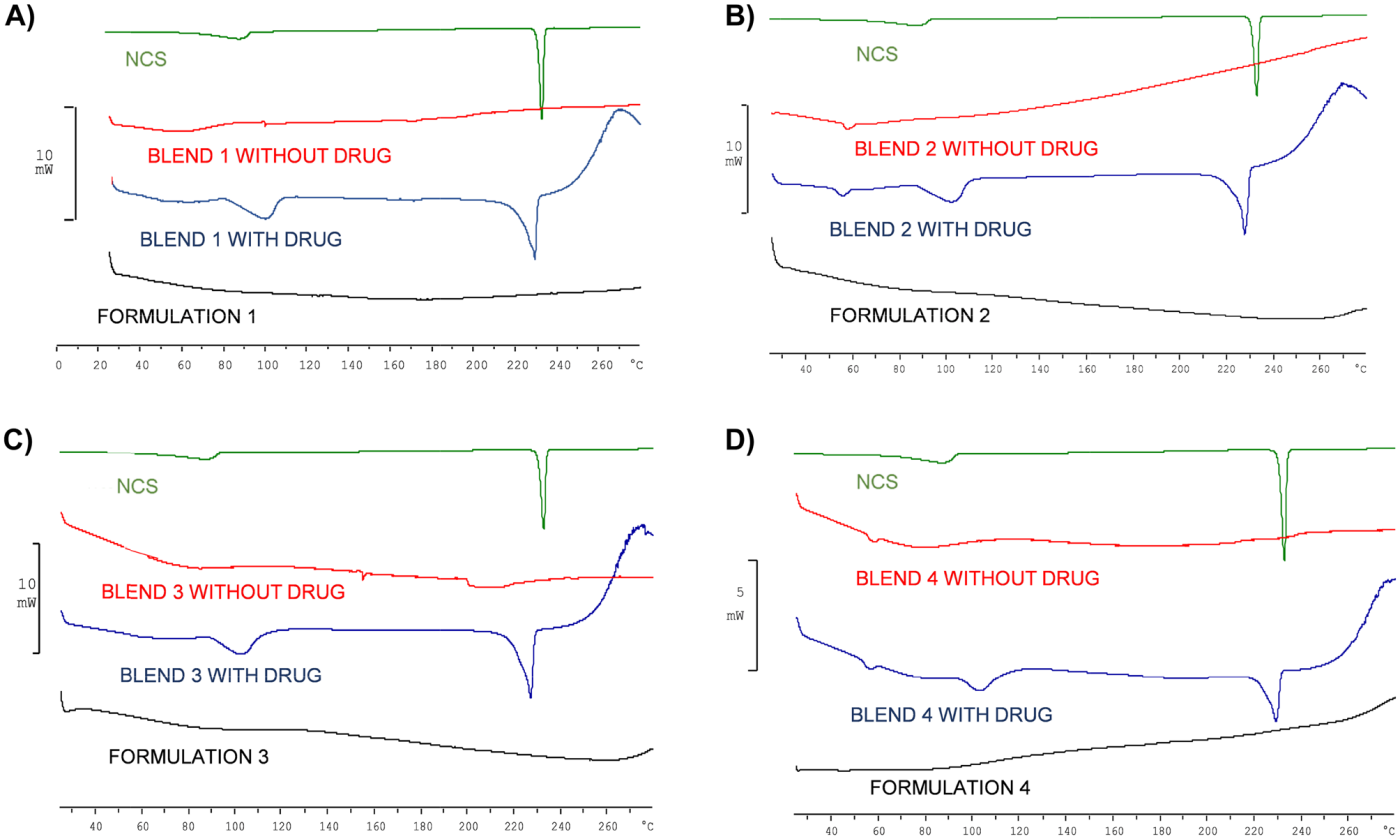
Thermograms of formulations 1 **A**), 2 **B**), 3 **C**), 4 **D**), each compared with the thermograms of the NCS and the respective blends with and without drug

A second test to verify this condition was performed by DSC. NCS shows a strong endothermic peak at 230 °C, descriptive of its crystalline nature (Fig. 9). A smaller endothermic peak is present in the thermogram of the physical blend with the drug. In contrast, the peak at 230 °C is completely absent in the thermogram of all formulations, probably due to amorphization of the drug during the printing phase and/or the formation of an inclusion complex between the NCS and HP-β-CD.

**Fig. 10:**
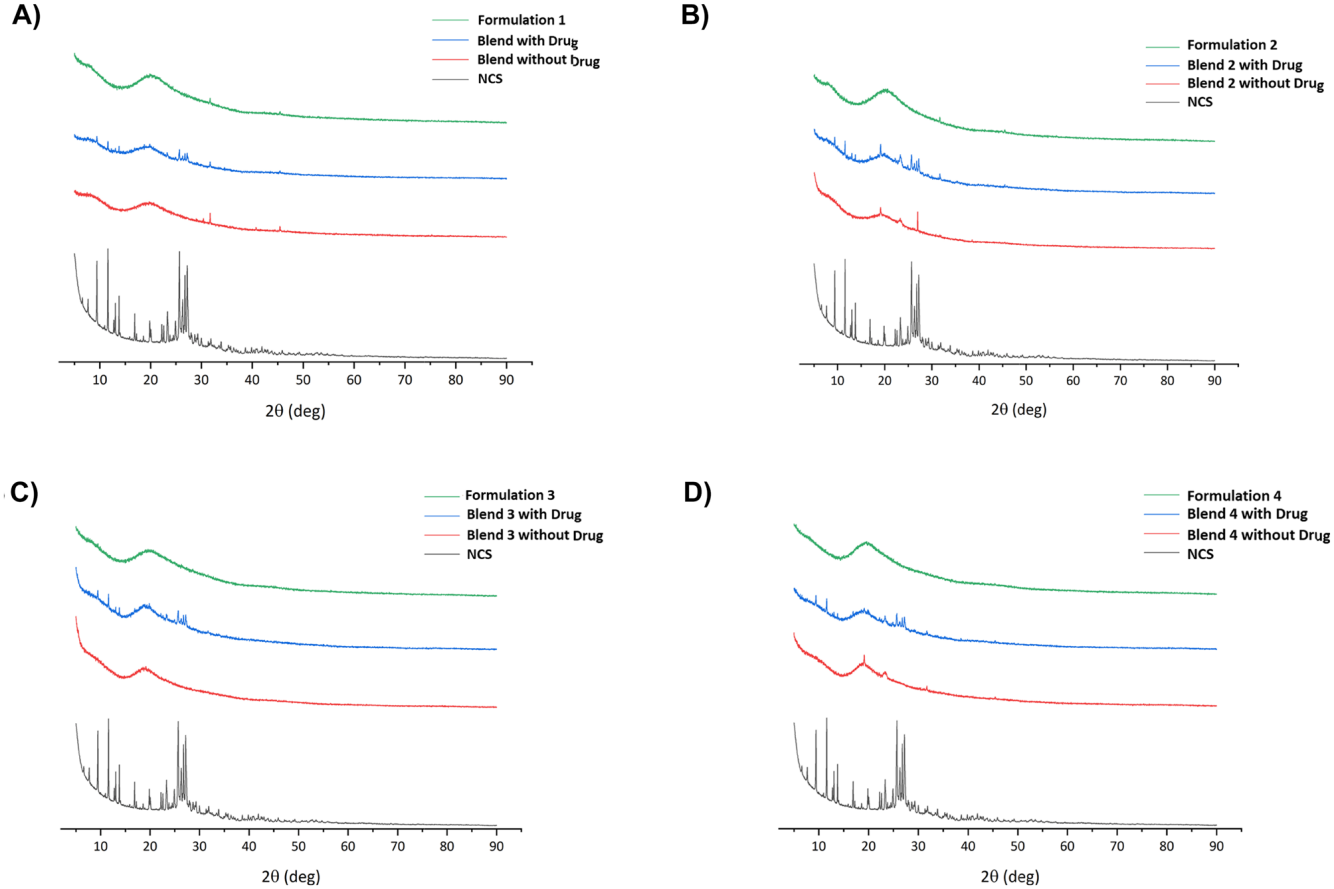
Diffractograms of the formulations 1 **A**), 2 **B**), 3 **C**), 4 **D**), each compared with the diffractograms of the NCS and the respective blends with and without drug

In Fig. 10 the powder diffraction patterns of NCS, blend with and without drug and formulations, are reported for each compound under investigation. The sharp peaks present in the pure NCS powder diffraction pattern indicate its crystallinity, while their complete disappearance in the patterns of formulations, clearly shows the NCS amorphization during the printing phase. This phenomenon, already pointed out by FT-IR and DSC results is confirmed by the PXRD analysis.

TGA analysis was conducted on both blend 4 and formulation 4. The choice of these samples was determined by the need to study the behavior of all the used excipients. The results obtained (Figure S1) for mixture and formulation 4 described a perfect sigmoidal pattern, confirming the stability of all components at the printing temperature of 180 °C. In addition, Figure S1 reported the first derivatives of the obtained values, indicating that the maximum peak of complete degradation of both the powder mixture and the formulation occurred at 300 °C, thus excluding any possible degradation during the printing phase.

To assess the stability of NCS during the printing process, HPLC analyses were carried out on a sample of NCS left for 15 min at 180 °C (printing time and extrusion temperature for one tablet) and on formulation 4 after complete solubilization. The chromatograms reported in Figure S2 show no sign of drug degradation, with only one NCS peak with a retention time of 6.1 min. We could therefore confirm that the thermal and mechanical stress induced by the printing process does not interfere with the stability of the drug.

Further confirmation of the stability of the drug was obtained by mass spectroscopy analysis performed on NCS kept at 180 °C for 15 min. Single quadrupole scanning in negative ion mode of the NCS confirmed that the main ion was the drug (m/z ion 326.97, [M]− H+) (data not shown).

### Dissolution testing of printed tablets

**Fig. 11:**
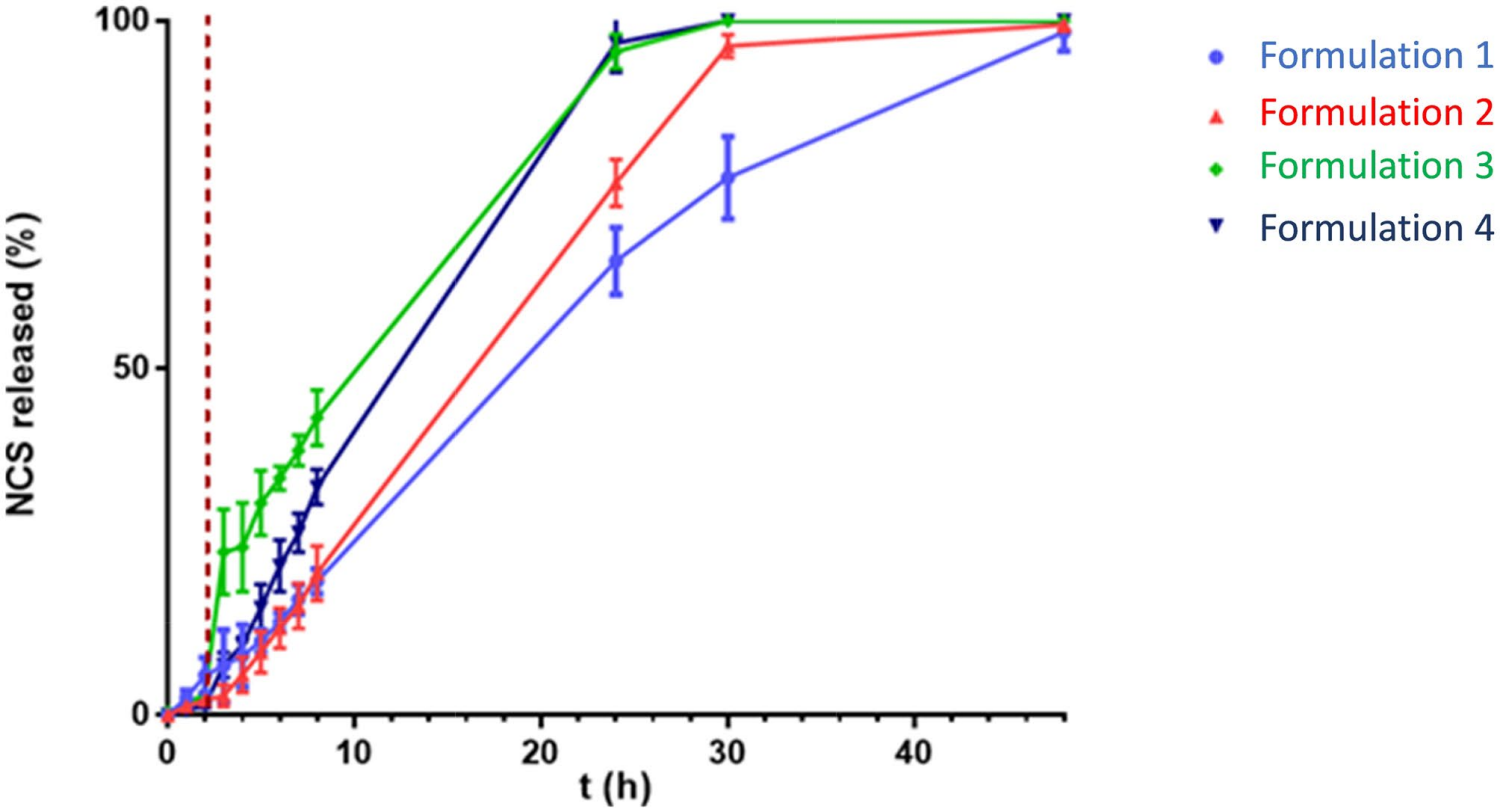
Dissolution profiles in gastric (2 h) and enteric (46 h) fluid of formulations

Dissolution studies of tablets obtained by direct extrusion of the four blends were performed for 48 h (Fig. 11). NCS release was studied in simulated gastric fluid for the first two hours and in simulated enteric fluid for the remaining hours. All formulations enhanced the dissolution profile of the pure drug, offering further confirmation of the advantage of this technique, which induced the loss of the drug's crystalline network during the printing process. Although at different times, all printed tablets showed a sustained and complete drug release within 48 h. In fact, HP-β-CD-containing formulations (3 and 4) achieved 100% NCS release in 24 h. In contrast, formulations 1 and 2 released 65% and 70% of NCS at 24 h, respectively, achieving complete drug release at 48 h. The different release profile of the tablets could be related to the different concentration of HPMC that constitutes them. In fact, the HPMC interacting with the aqueous fluid led to the formation of a gel layer, which acts as a diffusion barrier that counteracted drug release [43]. Several studies have been found in the literature concerning the behavior of HPMC when it is placed in contact with aqueous fluids [43]. Palugan et al. demonstrated the previously described phenomenon and provided as a potential solution the use of cellulolytic products capable of degrading the HPMC matrix more rapidly. The thickness of this layer may depend on the concentration of the polymer, in formulations 1 and 2 was present in higher concentrations than tablets containing HP-β-CD, causing a NCS release slower, but within 48 h. The high hydrophilicity of HP-β-CD and the ability of HPMC to stabilize the NCS/HP-β-CD complex in solution [26, 31] justify the improved release profile obtained from formulations 3 and 4. In addition, to produce sustained release tablets, a concentric filling pattern was selected in the set printing parameters. In fact, Obein et al. examined the influence on release profiles of four different filling patterns, showing that tablets with tri-hexagonal or concentric fillings had a longer release when compared to tablets with different filling geometries but the same composition [44]. In addition, they had shown that the selection of appropriate excipients affects the type of formulation that can be obtained, demonstrating that the use of HPMC allows the development of sustained-release tablets. Plotting the data of cumulative releases vs. time, for the first 8 h, obtained straight lines with R2 ˃ 0.96 for all tablets, suggesting that the release profiles follow kinetics of order 0.

The results obtained confirm the amorphization of the drug and the formation of a ternary inclusion complex between HP-β-CD, HPMC and NCS, inducing an improvement on drug loading and release.

### Stability studies

The stability of NCS in printed tablets was evaluated for 3 months (storage parameters 25 °C, 60% RH). The results highlighted that the amorphous state of the NCS, achieved following the extrusion process, is maintained and no signs of drug degradation were detected (data not shown).

## Conclusions

In this study, the feasibility of tablets containing NCS, HPMC and HP-β-CD by single-screw DPE 3DP has been proven. All the printed tablets showed good mechanical and physical features. By means of the *in vitro* dissolution studies, the results showed for all printed tablets a sustained release of NCS for 48 h, whereas for the tablets containing HP-β-CD the release was faster. HP-β-CD was used for the first time for direct powder extrusion using DPE, further improving the solubility of NCS and its release profile. This point has underlined the huge versatility and possible applicability of this innovative technique in the field of personalized therapy. The solid-state characterization study, using different techniques, revealed the loss of the crystalline state of the drug and a homogeneous distribution of the latter in the polymer matrix. In addition, through a stability study the drug content within the printed tablets was found to be stable for up to 3 months. Moreover, by using this innovative printer, 3DForMe®, which allows the direct extrusion of powders, it was possible to generate tablets, overcoming the limitations of filament production associated with FDM.

## Supplementary information

Below is the link to the electronic supplementary material.

Supplementary file1 (DOCX 150 KB)

## Data Availability

Not applicable.
