# Supplementary material for: Direct cyclodextrin-based powder extrusion 3D printing for one-step production of the BCS class II model drug niclosamide
Source: Drug Deliv Transl Res. 2022 Feb 9;12(8):1895–910. doi: 10.1007/s13346-022-01124-7 (PMC9242976; doi:10.1007/s13346-022-01124-7)
Supplement: Supplementary file 1 — Supplementary file1 (DOCX 150 KB) [file 13346_2022_1124_MOESM1_ESM.docx]

**SUPPLEMENTARY INFORMATION**

Direct cyclodextrin based powder extrusion 3D printing for one-step production of the BCS Class II Niclosamide

| Monica Pistone^1^; Giuseppe Francesco Racaniello^1^; Ilaria Arduino^1^; Valentino Laquintana^1^; Antonio Lopalco^1^; Annalisa Cutrignelli^1^; Rosanna Rizzi^2^; Massimo Franco^1^; Angela A. Lopedota^1, *^; Nunzio Denora^1, *^ |
| --- |
| ^1^ Department of Pharmacy – Pharmaceutical Sciences, University of Bari “Aldo Moro”, Orabona St. 4, 70125 - Bari, Italy  ^2^ Institute of Crystallography-CNR, Amendola St. 122/o, 70126 – Bari, Italy  *Corresponding authors: Prof. Nunzio Denora e-mail: [nunzio.denora@uniba.it](mailto:nunzio.denora@uniba.it) ; Prof.ssa Angela A. Lopedota e-mail: [angelaassunta.lopedota@uniba.it](mailto:angelaassunta.lopedota@uniba.it) |
| Materials and Methods1.1. TGA analysis The TGA analysis were performed on Blend 4 using PerkinElmer Thermogravimetric Analyzer Pyris 1 TGA. Sample (9 mg) was placed in platinum pans and was then heated from 30 to 600 °C using 5 °C/min as a heating rate. The thermal decomposition (or degradation) profile was analysed using Pyris™ software version 11 The experiments were carried out under nitrogen gas flow of 20 mL/min. |

**
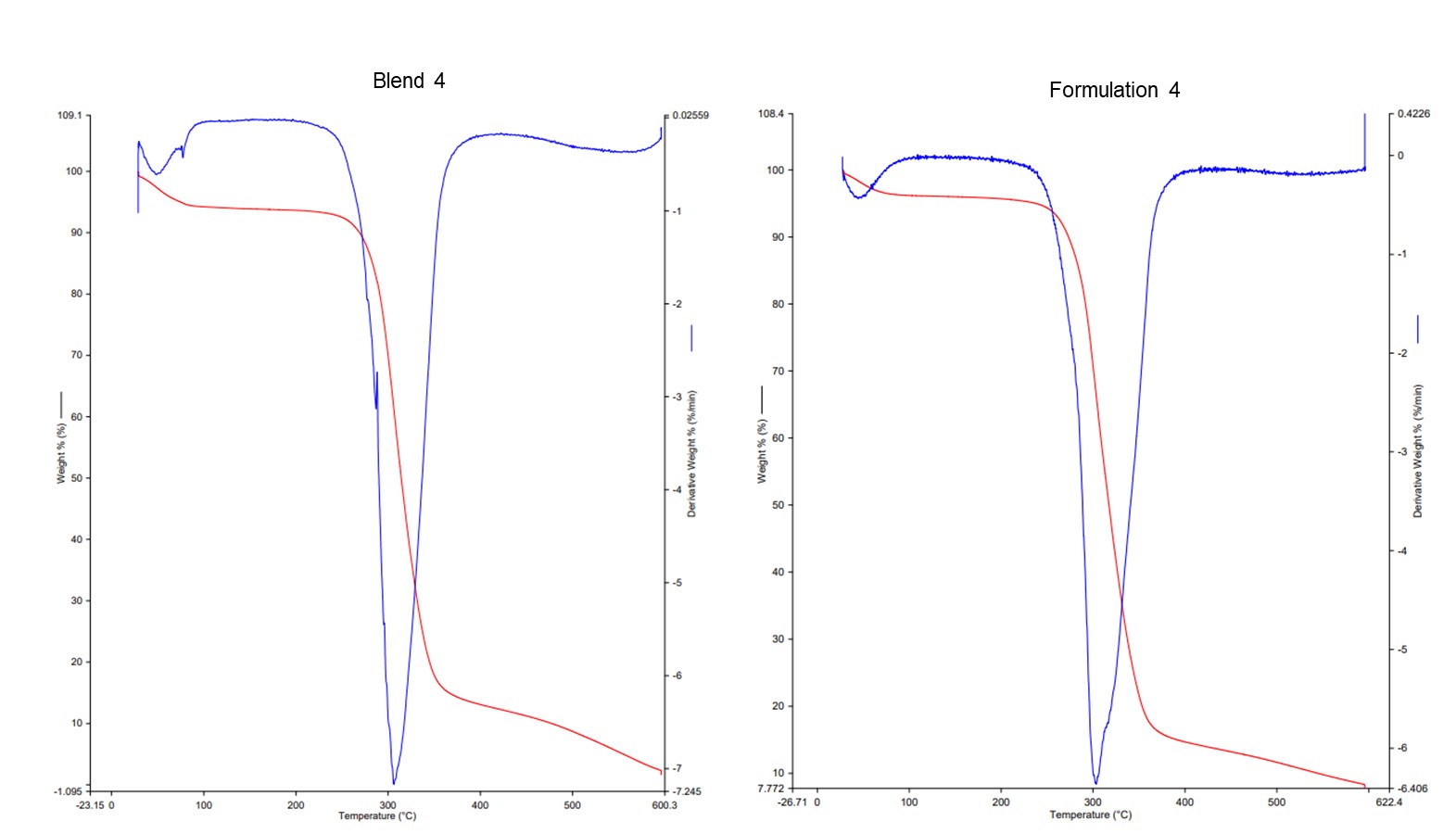
S1.** Thermogravimetric Analysis (TGA) of Blend 4 and Formulation 4.

#### 1.2. Qualitative Analysis of NCS by HPLC

To qualitatively assess the presence of not degraded NCS during the printing process, HPLC analyses were conducted. A sample of NCS maintained at 180 °C for 15 min and samples derived from the solubilisation of formulation 4 (see below) were also analysed. The investigations were performed with an Agilent 1260 Infinity Quaternary LC System equipped with an Agilent variable wavelength UV detector, a Rheodyne injector (Rheodyne, Model 7725i) equipped with a 20 µL loop and a OpenLAB CDS ChemStation software (Agilent, Santa Clara, CA). A ZORBAX Eclipse plus C18 column (4.6x250 mm 5-Micron) was used as the stationary phase and maintained at 30 °C for the duration of the analysis. The mobile phase consisted of methanol : phosphate buffer 1 mM (85:15 v/v) at pH 5.47 and was pumped at a flow rate of 0.7 mL/min. The wavelength selected for elution monitoring was 334 nm. A calibration line (R² = 0.9998) was obtained by analysing a range of concentrations between 0.05 and 0.005 mg/ml.

#### 1.3. Mass spectroscopy

Mass spectrometry was performed using Agilent 6530 accurate mass Q-TOF. Mass spectra were achieved in negative (ESI^-^).


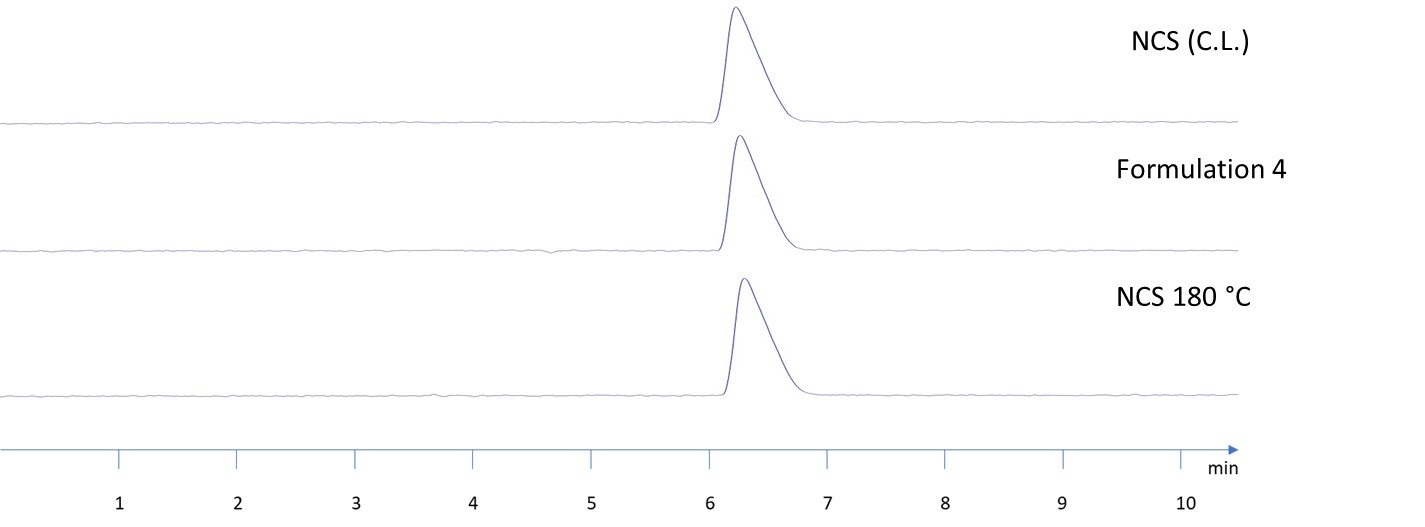


**S2**. Chromatograms for NCS (Calibration line), Formulation 4, and NCS placed at 180 °C for 15 minutes.
